# Applying genome-wide gene-based expression quantitative trait locus mapping to study population ancestry and pharmacogenetics

**DOI:** 10.1186/1471-2164-15-319

**Published:** 2014-04-29

**Authors:** Hsin-Chou Yang, Chien-Wei Lin, Chia-Wei Chen, James J Chen

**Affiliations:** 1 Institute of Statistical Science, Academia Sinica, No 128, Academia Road, Section 2, Nankang, Taipei, Taiwan; 2 School of Public Health, National Defense Medical Center, Taipei, Taiwan; 3 National Center for Toxicological Research, Food and Drug Administration, Little Rock, Arkansas, USA

**Keywords:** Gene-based approach, Expression quantitative trait locus (eQTL), Partial least squares (PLS), Ancestry-informative marker (AIM), Pharmacogenetics, Adverse drug reaction, Drug response, Drug biotransformation

## Abstract

**Background:**

Gene-based analysis has become popular in genomic research because of its appealing biological and statistical properties compared with those of a single-locus analysis. However, only a few, if any, studies have discussed a mapping of expression quantitative trait loci (eQTL) in a gene-based framework. Neither study has discussed ancestry-informative eQTL nor investigated their roles in pharmacogenetics by integrating single nucleotide polymorphism (SNP)-based eQTL (s-eQTL) and gene-based eQTL (g-eQTL).

**Results:**

In this g-eQTL mapping study, the transcript expression levels of genes (transcript-level genes; T-genes) were correlated with the SNPs of genes (sequence-level genes; S-genes) by using a method of gene-based partial least squares (PLS). Ancestry-informative transcripts were identified using a rank-score-based multivariate association test, and ancestry-informative eQTL were identified using Fisher’s exact test. Furthermore, key ancestry-predictive eQTL were selected in a flexible discriminant analysis. We analyzed SNPs and gene expression of 210 independent people of African-, Asian- and European-descent. We identified numerous cis- and trans-acting g-eQTL and s-eQTL for each population by using PLS. We observed ancestry information enriched in eQTL. Furthermore, we identified 2 ancestry-informative eQTL associated with adverse drug reactions and/or drug response. Rs1045642, located on *MDR1*, is an ancestry-informative eQTL (*P* = 2.13E-13, using Fisher’s exact test) associated with adverse drug reactions to amitriptyline and nortriptyline and drug responses to morphine. Rs20455, located in *KIF6*, is an ancestry-informative eQTL (*P* = 2.76E-23, using Fisher’s exact test) associated with the response to statin drugs (e.g., pravastatin and atorvastatin). The ancestry-informative eQTL of drug biotransformation genes were also observed; cross-population cis-acting expression regulators included *SPG7*, *TAP2*, *SLC7A7*, and *CYP4F2*. Finally, we also identified key ancestry-predictive eQTL and established classification models with promising training and testing accuracies in separating samples from close populations.

**Conclusions:**

In summary, we developed a gene-based PLS procedure and a SAS macro for identifying g-eQTL and s-eQTL. We established data archives of eQTL for global populations. The program and data archives are accessible at http://www.stat.sinica.edu.tw/hsinchou/genetics/eQTL/HapMapII.htm. Finally, the results from our investigations regarding the interrelationship between eQTL, ancestry information, and pharmacodynamics provide rich resources for future eQTL studies and practical applications in population genetics and medical genetics.

## Background

In the post-genome era, numerous genetic variations such as single nucleotide polymorphism (SNP) and rare variant have been discovered in international genomic projects such as the International HapMap Project [1-5], the ENCODE Project [6-8], and the 1000 Genomes Project [9,10]. The speedy and extensive discovery of genetic markers has facilitated not only a deeper understanding of the genomic makeup, but also an increased resolution for disease gene mapping. Genome-wide association studies have successfully identified thousands of genetic marker loci associated with downstream phenotypes, including disease susceptibility and quantitative traits [11-15]. However, a considerable proportion of the identified genetic loci are not located in protein-coding regions, suggesting that these genetic variants do not influence phenotypes through a change of gene function [16,17].

An expression quantitative trait locus (eQTL) is a genetic variation that regulates gene expression levels through a cis-regulatory (i.e., local regulation) and/or trans-regulatory (i.e., distant regulation) mechanism [18,19]. The importance of eQTL for an elucidation of disease etiology has been thoroughly documented [20-22]. Recent studies have revealed an enrichment of eQTL in the identified association signals, implying that gene regulation can be used to explain one of the major mechanisms regarding how genetic variants contribute to phenotypes [16,17,20,23]. The mechanism of eQTL regulation functions as a bridge between upstream genetic variation and downstream phenotypes; eQTL can control the transcript expression of functional genes through DNA binding, mRNA splicing, and noncoding RNA expression in transcriptional regulation, thereby conferring the alterations of downstream phenotypes [17].

Previous eQTL mapping studies have interconnected DNA-level markers and mRNA-level markers based on the various sources of human sample materials including blood [21], cell lines [24-27], and tissues [21,22,28] from the general population [24,25] or disease groups [20,29,30]. Several public eQTL databases for human genomic studies were established, including Genevar (http://www.sanger.ac.uk/resources/software/genevar/) [31], GTEx (http://www.ncbi.nlm.nih.gov/gtex/GTEX2/gtex.cgi) [32], seeQTL (http://www.bios.unc.edu/research/genomic_software/seeQTL/) [33], and the eQTL Browser at the Pritchard Lab (http://eqtl.uchicago.edu/cgi-bin/gbrowse/eqtl/). In addition, a number of studies have focused on mapping eQTL for non-human species such as yeast [34,35], Drosophila [36,37], and mice [38,39].

Most of the available studies have discussed eQTL mapping in a simple scenario in which they only modeled the relationship between single gene expression and a single SNP at a time [20,24,26,28,31,39-45]. In this simple case, an eQTL regulation can be evaluated through examining whether gene expression levels differ with respect to 2 alleles. Two-point linkage analysis [21,24] and single-locus association analysis [25,26] were performed to identify locus-based eQTL based on SNPs from low-density and high-density genotyping platforms, respectively. These studies have sketched the relationship of gene expression and eQTL but could be further improved by incorporating information from inter-marker correlation such as linkage disequilibrium in proximal SNPs and genetic interactions in distant SNPs.

Recent studies turned to correlate single gene expression with multiple SNPs simultaneously, whereby SNPs were chosen into a final model using sophisticated procedures of variable selection. Different statistical models, including analysis of variance [46], linear regression [35], and their generalizations such as penalized regression [47], have been considered before. These studies have gained a higher statistical power in detecting eQTL as well as increased the proportion of the variation of gene expression explained by multiple eQTL. However, the gene expression variation is still not thoroughly identified based on the findings of previous eQTL studies.

With the advent of microarray and sequencing technologies, the amount of high-resolution gene expression and SNP genotype data available has increased. Microarray data was used in this study. In a gene-expression microarray such as the Illumina’s gene expression bead chips [48] and Affymetrix’s gene expression gene chips [49], beads or probes are designed to measure the expression levels of multiple transcripts by targeting various transcriptional regions of a gene. In a SNP microarray such as Illumina’s SNP bead chips [50,51] and Affymetrix’s SNP gene chips [52,53], multiple probes are interrogated to measure the fluorescence intensities of SNP probe sequences and used to call the genotypes of SNPs. All transcript probes and all SNP genotypes targeting the genomic regions in a same gene can be simultaneously considered to gain a complete understanding regarding the interrelationship of gene expression and SNPs when mapping the eQTL that regulate the gene. This necessity motivated us to jointly correlate multiple transcript expression levels for a gene with multiple SNPs in the same gene.

This study used gene-based eQTL mapping. The concept of a gene-based association test has been broadly applied to genome-wide association studies [54-58], pharmacogenomics studies [59,60], and genetic diversity studies [61]. Gene-based analysis has become popular in genomic research because of its superior biological and statistical properties compared with those of a single-locus analysis. The strengths of a gene-based method include the following: 1) a higher power for the detection of minor-effect genes is gained by considering a joint effect of multiple markers; 2) the multiple-testing problem is alleviated by reducing the number of statistical tests; 3) an effect of locus heterogeneity is diluted and the results are more reproducible by analyzing and inferring an entire gene instead of a single marker; and 4) the results provide a direct interpretation in linking the association signals to genes [56,62]. Moreover, because a gene itself is a natural biological unit in genomes, genes provide a natural and feasible unit for forming neighboring SNPs as a marker set. To our knowledge, our study is the first genome-wide eQTL mapping study that jointly models multiple transcript probes and multiple SNP markers in a gene-based concept.

This study used a gene-based partial least squares (PLS) method to correlate the multiple transcript probes and multiple SNP markers. The original PLS method had been applied to gene expression data analysis for more than 10 years [63-65] and started to apply to genome-wide association study in the past few years [66-68]. However, the original PLS method did not consider gene information. This study developed a gene-based PLS that incorporated gene information into the original PLS to connect multiple transcript probes and multiple SNP markers in a genome-wide eQTL mapping study.

In addition to characterizing the distribution of eQTL in human genomes, this study also elucidated applications of ancestry-informative eQTL in population genetics and pharmacogenetics studies. Neither study has discussed ancestry-informative eQTL nor investigated their roles in pharmacogenetics before. In population genetics, ancestry-informative marker (AIM) is a type of genetic information marker that can be used for tracing the ancestral ethnicity of people. AIMs can be used to estimate the population indices such as allele frequency, genetic diversity, population differentiation, and admixture proportions for a characterization of the genetic background of study populations [69-74]. In addition, AIMs can also been used to infer ancestral or continental origin for criminal or victim identification in forensic sciences [70,75,76]. Short tandem repeat polymorphism markers [77,78], SNP markers [71,79-82], gene expression [82], and other types of biomarkers [83] have been employed for this purpose. This study especially interested in the capacity of eQTL for this purpose because identification of ancestry-informative eQTL can explain the regulatory mechanisms of genes and might correlate to protein product and functional changes in different ethnic populations.

This study also established panels of AIMs by using eQTL. In this study, data from 4 HapMapII populations were analyzed. Obtaining perfect AIM panels that can distinguish samples among the continental populations such as Africans, Caucasians and Asians [82] was not unexpected, but distinguishing samples from closely related populations, such as those from the Chinese and Japanese in this study was challenging. Therefore, we focused on classification analysis for these 2 relatively proximal Asian populations. If the method is effective, it can be further applied to study populations from much closer lineages. No studies have attempted to establish panels of AIMs by using eQTL.

Ancestry-informative eQTL are also useful in pharmacogenetics. In pharmacogenetics studies, genetic loci that influence adverse drug reactions, drug responses, and drug biotransformation have been identified and investigated [84,85]. Population-based pharmacogenetic association studies have been carried out and successfully identified several relevant pharmacogenetic loci [86,87]. However, previous studies have also demonstrated the changes of adverse drug reactions and drug responses [88-90] and drug biotransformation [91,92] by genetic ancestry. Pharmacogenetic association study should be conducted carefully to avoid results confounded by genetic heterogeneity of populations. Ancestry-informative eQTL can be used to reduce the false-positive or false-negative findings in pharmacogenetic association studies [70,82,93]: 1) ancestry-informative eQTL can be used as a covariate in a pharmacogenetic association analysis for adjusting for genetic substructures as a result of reduced false positives (i.e., to diminish spurious association); and 2) ancestry-informative eQTL can be used to classify individuals with different genetic backgrounds into homogenous groups for analysis as a result of reduced false negatives (i.e., to increase true association).

Pharmacogenetic markers can be applied to predict adverse drug reaction and drug response for personalized medicine [94]. A famous example is the application of the *HLA-B1502* allele in carbamazepine-induced Stevens-Johnson syndrome [95]. Seizure patients who carry the *HLA-B1502* allele incur a severe adverse drug reaction if they take carbamazepine for the treatment of seizure. However, this allele is only prevalent in Asian populations, not in Caucasian populations. Other markers are required to predict adverse drug reaction in Caucasian populations. Taken as a whole, the identification of ancestry-informative eQTL can assist clinicians regarding drug delivery and gain insights into pharmacodynamics and pharmacokinetics mechanisms in populations, and can also be used for correcting for population stratification or admixtures in a genome-wide association study of pharmacogenetics. Integrative analysis of eQTL, ancestry information, and pharmacogenetics is critical and warrant an urgent investigation.

## Methods

### Sample materials

This study analyzed 210 independent people from the International HapMapII Project [1-4]. The people consisted of 30 married African couples from Yoruba in Ibadan (YRI), 30 married Caucasian couples of European-descent residing in Utah (CEU), and 90 Asian people, including 45 Han Chinese people in Beijing (CHB) and 45 Japanese people in Tokyo (JPT). The Epstein-Barr virus-immortalized lymphoblastic cell lines of the 210 HapMapII samples were obtained from Coriell Cells Repositories (http://ccr.coriell.org/). For information regarding the preparation of DNA samples for SNP genotyping experiments and RNA samples for gene expression experiments, please refer to the references [1-4,27,40].

### SNP genotyping and data pre-processing

All 210 samples were genotyped using an Affymetrix Human Mapping 500 K Set (Affymetrix Inc., Santa Clara, CA, USA) [1-4]. This SNP chip provided genotype data for 500,568 SNPs on 23 pairs of human chromosomes. The Bayesian Robust Linear Model with Mahalanobis Distance Classifier [96] was used for calling SNP genotypes. The SNP genotype data are publicly available on the International HapMap Project website (http://hapmap.ncbi.nlm.nih.gov/). The annotation of the SNPs on the Affymetrix Human Mapping 500 K was derived from the NetAffx annotation update 28 (version: dbSNP Build 128) that is publicly available on the Affymetrix website (http://www.affymetrix.com/).

**Figure 1:**
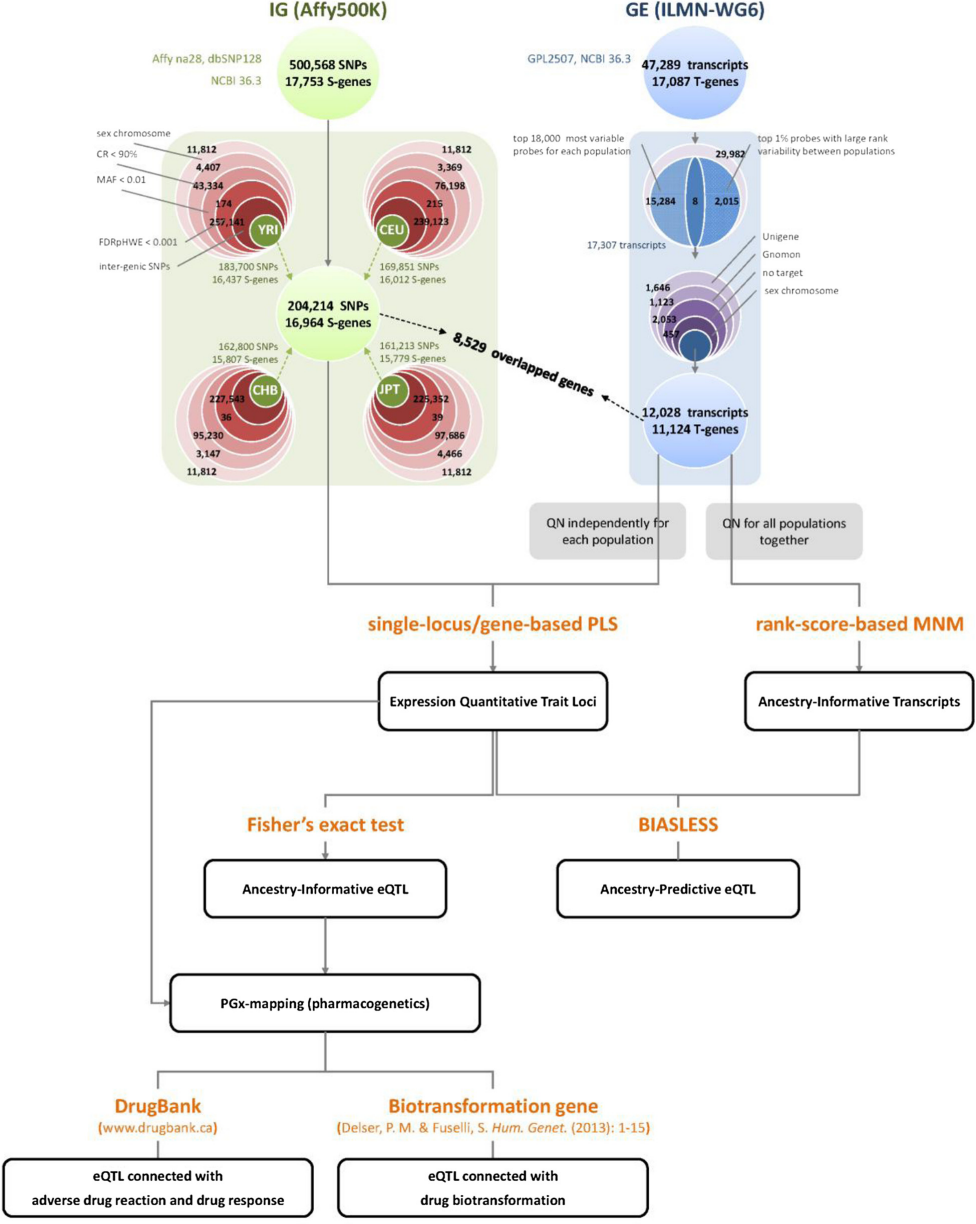
Flow chart of data pre-processing and ancestry-informative g-eQTL mapping.

The genotype data was preprocessed and analyzed for each of the 4 ethnic populations (YRI, CEU, CHB, and JPT). First, this study focused on autosomal SNPs; therefore, non-autosomal SNPs were removed. Second, poor-quality SNPs were removed if their genotype call rates were lower than 0.9, their minor allele frequencies were lower than 0.01, or they departed from Hardy-Weinberg equilibrium (HWE). Here, we claimed that a SNP violated the HWE if the P-value of a permutation-based HWE test [97] was lower than 0.001 after being adjusted using a false discovery rate procedure [98]. Finally, the inter-gene SNPs were removed from this gene-based eQTL mapping study. Throughout this paper, the genes containing those intra-gene SNPs are called “S-genes”, which means “sequencing-level genes”. The flow chart of the pre-processing of genotype data is described in the upper-left corner of Figure 1. In the subsequent SNP analyses, an additive coding of SNP genotype was used.

### Gene expression experiment and data pre-processing

The gene expression levels of the 210 HapMapII samples were measured using Illumina’s Sentrix Human-6 Expression BeadChip (Illumina Inc., San Diego, CA, USA) [27,40]. This bead chip provided 47,289 transcript probes targeting 17,087 genes for the human genome (4 control probes were removed in the beginning). The procedures used for the quantification and normalization of gene expression levels are described in the Supporting Online Materials [40]. The normalized gene expression data are publicly available in the Gene Expression Omnibus (GEO) database (http://www.ncbi.nlm.nih.gov/geo/) (Series accession number GSE6536). The annotation of gene expression probes on Illumina’s Sentrix Human-6 Expression BeadChip was derived from the GEO annotation (accession number GPL2507; version: UCSC HG 18) available in the GEO database (http://www.ncbi.nlm.nih.gov/geo/).

The gene expression data was preprocessed according to the procedures suggested in the original papers of this data set [27,40]. The main procedures are briefly described as follows. First, the top probes with the highest within-population variability were separately selected for the 4 populations (YRI, CEU, CHB, and JPT) separately. The probes that intersected in the lists of the 4 populations were collected. Second, all the probes were ranked by the within-population variability of gene expression in each population. The rank difference in each pair of populations was calculated and the top of the probes with the greatest rank differences was selected. Third, the union set of the 2 aforementioned probe lists was considered. Fourth, we focused on the biologically validated transcripts from the RefSeq database and removed the transcripts from the UniGene and Gnomon databases whose target genes were obtained from using algorithm predictions. Finally, we focused on autosomal transcripts; non-autosomal transcripts were removed. Throughout this paper, the genes targeted by transcript probes are called “T-genes”, which means “transcript-level genes”. The flow chart of the pre-processing of gene expression data is described in the upper-right corner of Figure 1.

### Gene-based eQTL mapping

We applied a gene-based PLS method to correlate the multiple transcript probes for a T-gene and multiple SNPs on an S-gene. Mapping for SNP-based eQTL becomes a special case if a PLS analysis is performed to correlate the transcript expression levels of genes with a single SNP. The analysis was performed using a SAS macro we developed, which was modified from the SAS procedure, PROC PLS (SAS Inc., Cary, NC, USA) (Additional file 1). The gene-based PLS method is also provided (Appendix). We implemented a 7-fold cross-validation procedure to calculate the root mean predicted residual sum of squares (PRESS) for each number of extracted genetic factors, and the number of extracted genetic factors that had the minimum root mean PRESS was chosen. To avoid over-fitting because of the inclusion of too many genetic factors in a model, the van der Voet’s test [99] was applied to determine a final model with a reduced number of extracted genetic factors. The final model had the least genetic factors with a root mean PRESS that was insignificantly larger than the model with the minimum root mean PRESS. A set of SNPs on an S-gene was defined as “gene-based eQTL” (g-eQTL) if the number of extracted genetic factors in the final model was greater or equal to 1, and the variation in the S-gene and T-gene accounted for by the factor(s) was non-zero. A g-eQTL is cis-regulatory if the S-gene where the g-eQTL is located and the T-gene that is regulated by the g-eQTL are the same gene; otherwise, the g-eQTL is trans-regulatory. The correlation between the g-eQTL (S-gene) and the regulated gene (T-gene) was measured using the coefficient of canonical correlation. The flow charts of an eQTL mapping, an identification of ancestry-informative eQTL associated with drug or drug biotransformation (described in the next subsection), and an identification of key ancestry-informative eQTL with a high predictive ability for ethnic groups (or termed as “ancestry-predictive eQTL”) (described after the next subsection) are summarized in the bottom of Figure 1.

### Identification of the ancestry-informative eQTL associated with adverse drug reactions and drug responses

A 3-step procedure was used to identify ancestry-informative eQTL associated with adverse drug reactions and drug responses. In the first step, ancestry-informative eQTL was identified. We collected the eQTL identified in our eQTL mapping analysis and examined whether they exhibited differential allelic distributions in the study populations by using Fisher’s exact test. In the analysis of the contingency table, the first variable was the allele type of an eQTL (2 allele types of a SNP) and the second variable was the ethnic groups being studied (4 populations: YRI, CEU, CHB, and JPT). An eQTL was claimed as ancestry-informative if after being adjusted using a false discovery rate procedure [98], the adjusted P-value of the Fisher’s test was smaller than 0.05. In the second step, we collected the SNPs associated with drugs. We collected all the SNPs reported to have adverse drug reactions (SNP-ADR) and the SNPs associated with drug responses (SNP-FX) in the DrugBank database (http://www.drugbank.ca/). Simultaneously, we also examined if those SNPs in pharmacogenetics used in this study were also interrogated by using the Affymetrix Human Mapping 500 K Set. In the final step, the ancestry-informative eQTL associated with adverse drug reactions and drug responses were obtained by taking the intersection of the ancestry-informative eQTL identified in the first step and the SNPs in pharmacogenetics collected in the second step. When we identified genome-wide eQTL associated with adverse drug reactions and drug responses, we chose the intersection of genome-wide eQTL and the SNPs associated with drugs.

### Identification of the ancestry-informative eQTL associated with drug biotransformation

First, we collected the eQTL identified in our eQTL mapping analysis. Second, we compiled the drug biotransformation genes reported in the literature [91]; they consisted of genes with encoding phases I and II drug-metabolism enzymes, the cytochrome P450 drug metabolizing enzymes, and the genes responsible for drug transporters, transcription factors, among others. Third, we extracted the cis-acting and trans-acting eQTL that regulated the drug biotransformation genes in our data. Finally, the identified eQTL that regulated the drug biotransformation genes were examined whether they were ancestry-informative by using Fisher’s exact test.

### Identification of the key ancestry-predictive eQTL

A 2-step procedure was used to identify key ancestry-predictive eQTL satisfying the following 2 conditions: 1) the eQTL can regulate ancestry-informative transcripts, and 2) the eQTL can distinguish samples from different populations. In the first step, we identified ancestry-informative transcripts, which were differentially expressed in the study populations. We adapted a rank-score-based multivariate nonparametric method (MNM) [100] for a gene-based association test to identify ancestry-informative transcripts. In the analysis, the response variables were multivariate gene expression levels of transcript probes for a T-gene and the independent variable was the ethnic groups of interest. The transcript probes for a T-gene were defined as ancestry-informative when the adjusted P-value of the MNM test was lower than 0.05 by using a false discovery rate procedure [98].

In the second step, we identified the key ancestry-predictive eQTL that regulated the ancestry-informative transcripts. We collected all the s-eQTL and g-eQTL associated with ancestry-informative transcripts identified in the first step. Incorporating a forward selection procedure, we then chose key ancestry-predictive eQTL from the collection of ancestry-informative eQTL by using statistical discriminant analysis, using BIASLESS software [82]. Here we focused on the discussion of the results in CHB and JPT populations because these closely related populations were more difficult to classify in the sample classification analysis compared to the other 2 distant populations, YRI and CEU. We performed classification analyses by using 3 eQTL sets: 1) the eQTL found in CHB or JPT (i.e., union), 2) eQTL found in CHB and JPT (i.e., intersection), and 3) eQTL found only in CHB and JPT (i.e., differences). The main statistical procedures in each classification analysis are described as follows.

We implemented a 10-fold cross-validation procedure. In each cross-validation, a flexible discriminant analysis was used to build a classification model with the highest testing accuracy by sequentially selecting the eQTL with the maximum increment of training accuracy in the eQTL list. The eQTL with the minimum within-population and between-population sum of squares ratio for genotypic values was selected if more than one eQTL had the same training accuracy. The procedure continued until the training accuracy reached 1.0 or the increment of training accuracy was less than 0.001. The model with the highest training accuracy was then used to classify individuals in the testing dataset and the testing accuracy was calculated. The previous steps were repeated until each of the 10 subsets of data had been analyzed as a testing dataset, resulting in 10 classification candidate models. Finally, among the 10 classification models, the model with the highest testing accuracy was selected as the optimal classification model.

## Results

### SNP data pre-processing

In this study, we started with a removal of 11,812 (11,812, 11,812, and 11,812) non-autosomal SNPs from the YRI (CEU, CHB, and JPT) population. Next, we sequentially removed 4,407 (3,369, 3,147, and 4,466) low-call-rate SNPs, 43,334 (76,198, 95,230, and 97,686) non-polymorphic SNPs, and 174 (215, 36, and 39) HWE-violated SNPs. We further removed 257,141 (239,123, 227,543, and 225,352) inter-gene SNPs. The final remains were 183,700 (169,851, 162,800, and 161,213) SNPs for our subsequent analysis. The union of the 4 SNP sets in the 4 study populations contained 204,214 distinct SNPs (the upper-left corner of Figure 1).

SNP-to-gene mapping was performed according to the physical positions of SNP and gene expression markers from the aforementioned SNP and gene annotations and the seq_gene.md file in NCBI 36.3. A SNP was assigned to all the genes if the SNP were mapped to multiple genes by annotations. In this study, 204,214 SNPs were mapped to 16,964 S-genes, which was the combination of 16,437 genes in YRI, 16,012 genes in CEU, 15,807 genes in CHB, and 15,779 genes in JPT (the upper-left corner of Figure 1).

### Gene expression data pre-processing

First, the top 18,000 probes with the highest within-population variability were selected in each of the 4 ethnic populations (YRI, CEU, CHB, and JPT). The intersection set of the 4 probe lists for highest within-population variability contained 15,292 probes. Second, in each population, the gene expression variability of probe was ranked and the top 1% of probes (473 probes) with the greatest variability of ranks was selected. Of the 2,838 probes with the greatest variability of ranks in 6 pairwise comparisons of the 4 populations, 2,023 were distinct probes. A total of 17,307 distinct probes were identified in the 2 aforementioned probe lists; in other words, 8 probes were overlapped in the 2 sources of probes. Of the 17,307 probes, we removed 2,769 non-RefSeq probes (1,646 probes from UniGene and 1,123 probes from Gnomon) and then removed 2,510 non-autosomal transcript probes (2,053 probes without chromosome information and 457 probes targeting genes located on sex chromosomes). Finally, 12,028 transcript probes for 11,124 T-genes were used for the subsequent analysis (the upper-right corner of Figure 1).

### Gene-based eQTL mapping

We performed gene-based eQTL mapping by analyzing 16,964 S-genes and 11,124 T-genes in the 4 study populations, where 8,529 genes overlapped. The analysis revealed 302, 235, 239, and 259 T-genes regulated by g-eQTL through a cis-acting mechanism in the YRI, CEU, CHB, and JPT populations, respectively. Twenty-five of the T-genes were simultaneously identified in all of the 4 study populations: *APIP*, *C8orf32*, *CAPZA1*, *EFCAB2*, *ENTPD1*, *FAHD1*, *FAM119B*, *HLA-DQA1*, *LOC339804*, *LOC388335*, *MASTL*, *MED29*, *NT5C3L*, *PDPR*, *PKHD1L1*, *SERPINB10*, *SLFN5*, *SNX11*, *SPG7*, *SQSTM1*, *ST7L*, *TIPRL*, *TRIM4*, *TSGA10*, and *ZNF230*. The analysis revealed 205, 192, 193, and 193 cis-regulatory g-eQTL contained in the corresponding S-genes of the 25 T-genes in the YRI, CEU, CHB and JPT populations, respectively. The analysis also identified 11,094, 11,102, 11,110, and 11,117 T-genes regulated by g-eQTL through a trans-acting mechanism in the YRI, CEU, CHB, and JPT populations, respectively.

### Identification of the ancestry-informative eQTL associated with adverse drug reactions and drug responses

In the first step, we identified ancestry-informative g-eQTL. Based on 203,618 distinct g-eQTL (180,181, 165,080, 152,909, and 142,371 g-eQTL, derived from YRI, CEU, CHB and JPT, respectively), the Fisher’s exact tests identified 181,655 g-eQTL with a differential allelic distribution in 4 populations; therefore, these were ancestry-informative (159,458, 144,670, 133,429, and 124,072 g-eQTL from YRI, CEU, CHB, and JPT, respectively). Over 80% of the identified g-eQTL were ancestry informative. A similar pattern of ancestry information enrichment was also observed in s-eQTL.

In the second step, we collected the drug-associated SNPs reported in DrugBank. Fifty distinct SNPs related to adverse drug reactions (from the “SNP-ADR” table in DrugBank) and 25 distinct SNPs related to drug response (from the table of “SNP-FX” in DrugBank). Three SNPs (rs1045642, rs5030655, and rs3892097) were related to both adverse drug reactions and drug responses. Among the 72 distinct SNPs in pharmacogenetics, only 4 SNPs were interrogated in the Affymetrix Human Mapping 500 K Set used in this study. After excluding one SNP (rs1799853) without chromosome information and removing one SNP located on a sex chromosome (rs4825476), the remaining 2 SNPs were rs1045642 and rs20455.

We found that rs1045642 and rs20455 were ancestry-informative SNPs in the first step. In other words, whether or not ancestry information was considered in advance, this study identified rs1045642 and rs20455 as eQTL associated with adverse drug reactions and/or drug response. Rs1045642 is located in the ATP-binding cassette B1 gene (*ABCB1*), aliased as Multi-drug resistance gene 1 (*MDR1*) on the chromosome 7q21.12. Rs1045642 is an ancestry-informative eQTL (*P* = 2.13E-13, using Fisher’s exact test) associated with adverse drug reactions to amitriptyline and nortriptyline [101] and drug responses to morphine [102]. This eQTL regulates 7, 28, and 9 T-genes in YRI, CHB, and JPT, respectively. Rs20455, which is located in *KIF6* on the chromosome 6p21.2, is also an ancestry-informative eQTL (*P* = 2.76E-23, using Fisher’s exact test) associated with drug responses to pravastatin and atorvastatin [103]. This eQTL regulates 15, 45, 9, and 45 T-genes in YRI, CEU, CHB, and JPT, respectively.

### Identification of the ancestry-informative eQTL of drug biotransformation genes

Among the 143 drug biotransformation genes collected from the literature [91], only 139 drug biotransformation genes had data available in the Illumina’s Sentrix Human-6 Expression BeadChip. We identified several s-eQTL that regulated drug biotransformation genes. In CEU, 5 drug biotransformation genes (*CAT*, *CYP4F2*, *SLCO1B3*, *SPG7*, and *TAP2*) were cis-regulated by 11 s-eQTL and 76 drug biotransformation genes were trans-regulated by 21,213 s-eQTL. In YRI, 5 drug biotransformation genes (*ABCC4*, *GSTO1*, *PTGS1*, *SPG7*, and *ADK*) were cis-regulated by 22 s-eQTL and 76 drug biotransformation genes were trans-regulated by 20,981 s-eQTL. In CHB, 5 drug biotransformation genes (*TAP2*, *SLC7A7*, *SPG7*, *CYP4F2*, and *CBR3*) were cis-regulated by 8 s-eQTL and 76 drug biotransformation genes were trans-regulated by 20,314 s-eQTL. In JPT, 3 drug biotransformation genes (*TAP2*, *SLC7A7*, and *SPG7*) were cis-regulated by 9 s-eQTL and 76 drug biotransformation genes were trans-regulated by 17,659 s-eQTL. Moreover, we found that all of the identified cis-acting s-eQTL that regulated drug biotransformation genes were ancestry-informative. Among the identified trans-acting s-eQTL that regulated drug biotransformation genes, 18,583 (87.60%), 18,521 (88.28%), 17,717 (87.22%), and 15,271 (86.48%) of which were ancestry-informative in CEU, YRI, CHB, and JPT, respectively.

We also identified g-eQTL that regulated drug biotransformation genes. In CEU, 3 drug biotransformation genes (*CAT*, *SLCO183*, and *SPG7*) were cis-regulated and 76 genes were trans-regulated by 1,964 S-genes (16,050 g-eQTL). In YRI, 3 drug biotransformation genes (*GSTO1*, *PTGS1*, and *SPG7*) were cis-regulated and 76 genes were trans-regulated by 1,634 S-genes (12,783 g-eQTL). In CHB, 3 drug biotransformation genes (*TAP2*, *SPG7*, and *CBR3*) were cis-regulated and 76 genes were trans-regulated by 2,113 S-genes (18,506 g-eQTL). Finally, in JPT, 3 drug biotransformation genes (*TAP2*, *SLC7A7*, and *SPG7*) were cis-regulated and 76 genes were trans-regulated by 1,803 S-genes (15,046 g-eQTL). We found that 35 (97.22%), 29 (96.67%), 15 (88.24%), and 22 (88%) cis-acting g-eQTL that regulated drug biotransformation genes were ancestry-informative in CEU, YRI, CHB, and JPT, respectively. Among the identified trans-acting g-eQTL that regulated drug biotransformation genes, 14,075 (87.69%), 11,206 (87.66%), 16,059 (86.78%), and 13,089 (86.99%) of which were ancestry-informative in CEU, YRI, CHB, and JPT, respectively.

### Identification of key ancestry-predictive g-eQTL

**Figure 2:**
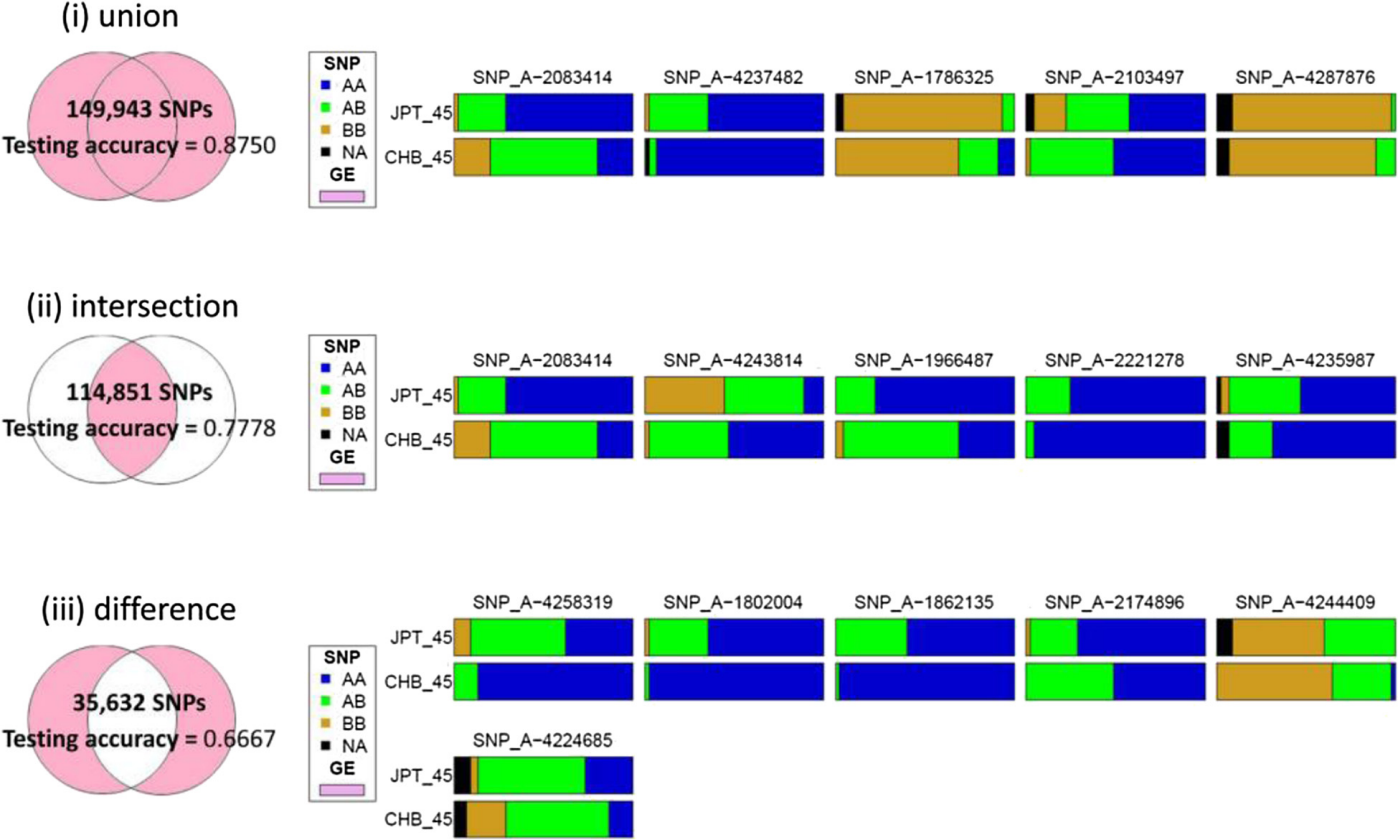
Results of classification analysis for identifying AIMs based on g-eQTL found in “CHB or JPT”, “CHB and JPT”, or “CHB-specific or JPT-specific”.

In the first step, we identified ancestry-informative transcripts. The MNM tests revealed 6,241 targeted T-gene differentially expressed in 2 proximal populations, CHB and JPT. All the cis-acting and trans-acting g-eQTL for the 6,241 ancestry-informative transcripts were collected. In CHB, 881 cis-acting g-eQTL on 112 S-genes and 146,421 trans-acting g-eQTL on 14,976 S-genes were identified. In JPT, 1,214 cis-acting g-eQTL on 132 S-genes and 133,439 trans-acting eQTL on 14,479 S-genes were identified. In summary, 114,851 g-eQTL on 13,714 S-genes were shared in both populations (i.e., intersection) and 149,943 g-eQTL on 15,436 S-genes occurred in CHB or JPT populations (i.e., union) (Figure 2). Moreover, 23,197 g-eQTL on 1,078 S-genes were CHB-specific and 12,448 g-eQTL on 644 S-genes were JPT-specific, and 3 SNPs overlapped in the 1,722 population-specific S-genes.

In the second step, we selected key ancestry-predictive g-eQTL from the sets of g-eQTL identified in “CHB or JPT”, “CHB and JPT”, or “CHB-specific or JPT-specific” through a classification analysis, using BIASLESS software. The 3 classification analyses selected 5, 5 and 6 ancestry-informative eQTL for separating individuals from the CHB and JPT populations with a testing accuracy of 0.8750, 0.7778, and 0.6667, respectively (Figure 2). In the analysis of “CHB or JPT”, the key ancestry-predictive eQTL were SNP_A-2083414 (rs7045959), SNP_A-4237482 (rs770576), SNP_A-1786325 (rs12637414), SNP_A-2103497 (rs7757158), and SNP_A-4287876 (rs12551120). In the analysis of “CHB and JPT”, the key ancestry-predictive eQTL were SNP_A-2083414 (rs7045959), SNP_A-4243814 (rs2976396), SNP_A-1966487 (rs1561296), SNP_A-2221278 (rs2074066), and SNP_A-4235987 (rs731952). In the analysis of “CHB-specific or JPT-specific”, the key ancestry-predictive eQTL were SNP_A-4258319 (rs10956197), SNP_A-1802004 (rs17807611), SNP_A-1862135 (rs4244011), SNP_A-2174896 (rs17644158), SNP_A-4244409 (rs1001021), and SNP_A-4224685 (rs7302554).

### Comparison of ancestry-informative g-eQTL and s-eQTL

We also implemented s-eQTL mappings and examined their performance in inferring population ancestry. In CHB, 177 cis-acting s-eQTL on 170 S-genes and 156,349 trans-acting s-eQTL on 15,615 S-genes were identified. In JPT, 212 cis-acting s-eQTL on 202 S-genes and 153,101 trans-acting s-eQTL on 15,507 S-genes were identified. In summary, 145,107 s-eQTL on 15,212 S-genes were shared in both CHB and JPT populations and 155,452 s-eQTL on 15,562 S-genes occurred in the CHB or JPT populations. Moreover, 5,743 s-eQTL on 2,734 S-genes were CHB-specific and 4,602 s-eQTL on 2,253 S-genes were JPT-specific.

**Figure 3:**
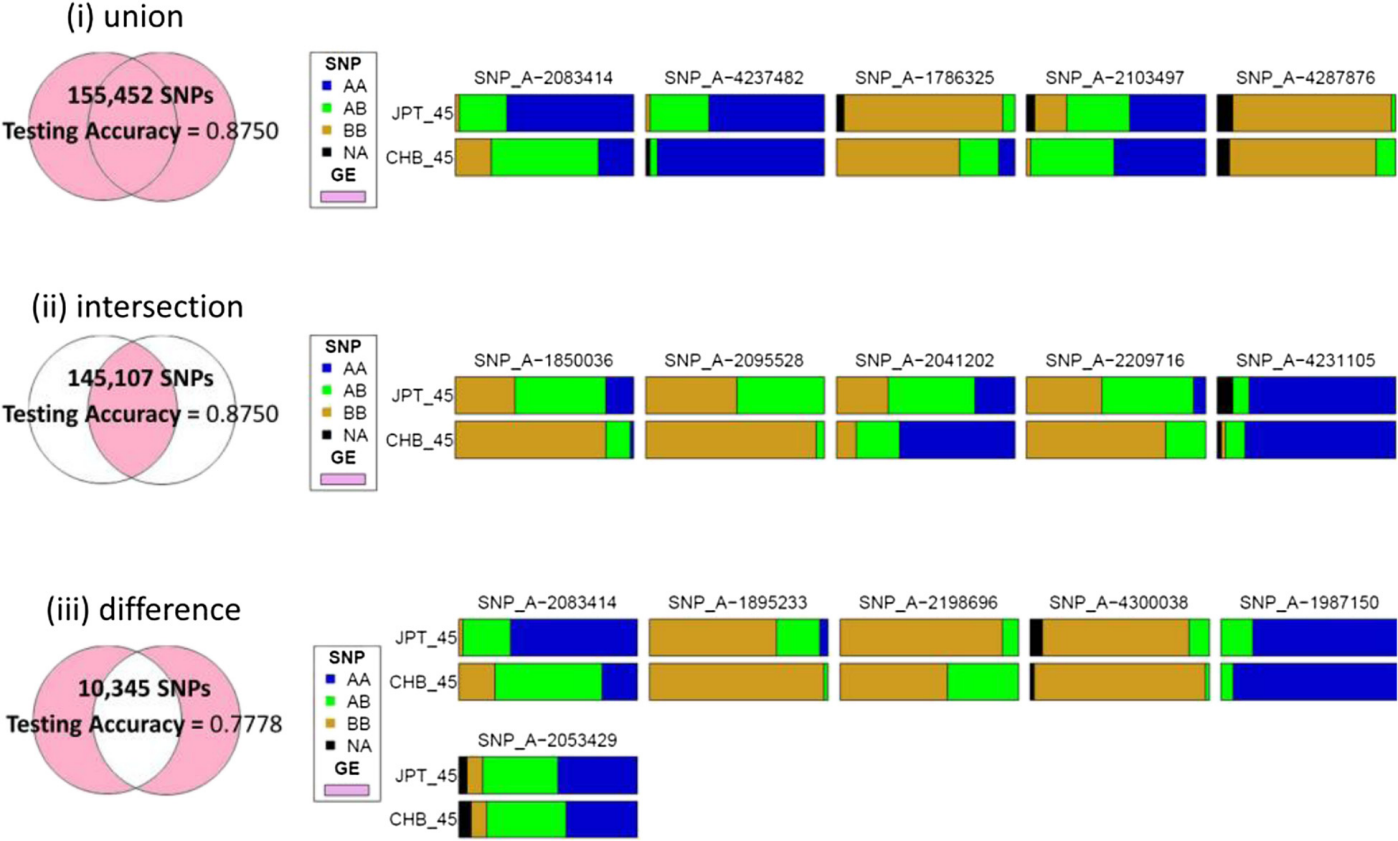
Results of classification analysis for identifying AIMs based on s-eQTL found in “CHB or JPT”, “CHB and JPT”, or “CHB-specific or JPT-specific”.

**Figure 4:**
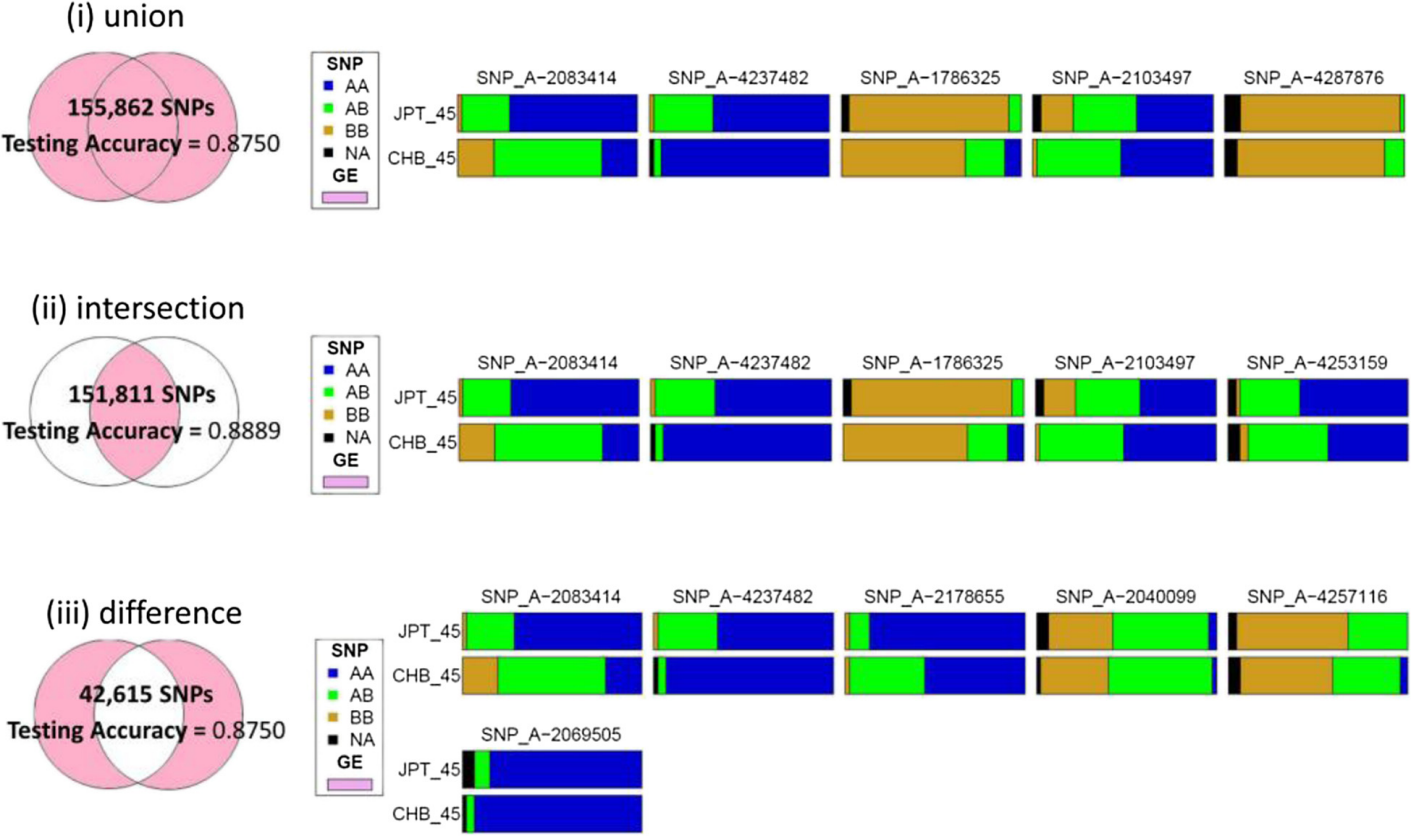
Results of classification analysis for identifying AIMs based on the combination of g-eQTL and s-eQTL found in “CHB or JPT”, “CHB and JPT”, or “CHB-specific or JPT-specific”.

In the sets of eQTL for “CHB or JPT”, “CHB and JPT”, and “CHB-specific or JPT-specific”, the numbers of overlapping g-eQTL and s-eQTL were 149,533, 108,147, and 3,362, respectively. Compared with the analysis based on g-eQTL only, if key ancestry-predictive eQTL were chosen from only s-eQTL, testing accuracy increased to 0.8750, 0.8750, and 0.7778 for “CHB or JPT”, “CHB and JPT”, or “CHB-specific or JPT-specific”, respectively (Figure 3). If key ancestry-predictive eQTL were chosen from a combination of g-eQTL and s-eQTL, the testing accuracy further increased to 0.8750, 0.8889, and 0.8750 (Figure 4).

### The SAS macro for eQTL mapping using a gene-based PLS analysis

We provided a SAS macro for gene-based and SNP-based eQTL mapping (Additional file 1). The gene expression data were saved in separated files organized by T-genes and the genotype data of SNPs were saved as separated files by S-genes. For convenience, if the numbers of the transcripts in T-genes are the same, the gene expression data of those T-genes can also be arranged in the same file. Similarly, if the numbers of SNPs on S-genes are the same, the genotype data of those S-genes can be saved in the same file. In each data file of a T-gene, the first 2 columns are the T-gene name and sample ID followed by the gene expression data of multiple transcript probes in this T-gene. In each S-gene data file, the first 2 columns are the S-gene name and sample ID followed by the genotype data (0, 1, or 2) of multiple SNPs in this S-gene. The main command line of the SAS macro was the following:

$$\begin{array}{l} \%\mathrm{macro}\;\mathrm{gbPLS}(\mathrm{Tgene}=,\mathrm{Sgene}=,\mathrm{nProbe}=,\\ \;\mathrm{nSNP}=,\mathrm{Out}=) \end{array}$$

Users must specify the “filename of gene expression data of T-genes”, “the filename of genotype data of S-genes”, “the number of transcript probes in T-genes”, “the number of SNPs on a S-gene”, and “the output filename” in the command line after the symbol “=”. We also provided an illustrative example. In this example, we examined the association between the gene expression of 7 T-genes that each of them had 4 transcripts (Additional file 2) and genotype data of 43 S-genes that each contained 28 SNPs (Additional file 3). The gene expression data were saved using the filename “GE”. The genotype data were saved using the filename “SNP”. The outputs were saved using the filename “OUTPUT”. The command line for this example is written as follows:

$$\begin{array}{l} \%\mathrm{macro}\;\mathrm{gbPLS}(\mathrm{Tgene}=\mathrm{GE},\mathrm{Sgene}=\mathrm{SNP},\\ \;\mathrm{nProbe}=4,\mathrm{nSNP}=28,\\ \;\mathrm{Out}=\mathrm{OUTPUT}); \end{array}$$

Users can also run a SNP-based eQTL mapping using this SAS macro by providing a genotype file that contains the data of a single SNP.

### The eQTL data archives

The population-specific eQTL data archives for the 4 study populations (YRI, CEU, CHB, and JPT) were established in this study. To facilitate the search of eQTL results, 3 Excel files (Additional files 4, 5, and 6), that summarize the relationships between T-genes, S-genes, and eQTL, were prepared and are also accessible at the website (http://www.stat.sinica.edu.tw/hsinchou/genetics/eQTL/HapMapII.htm).

## Conclusion and discussion

In conclusion, our research makes several contributions for future eQTL studies. Regarding methodology, we augment a gene-based PLS procedure into the strategies for mapping eQTL. Moreover, we also provide a SAS macro for an efficient identification of s-eQTL and g-eQTL. In biology, our eQTL mapping study provides detailed cis-acting and trans-acting connections between T-genes and S-genes/loci. The results reveal the relevance and complexity of gene regulation in the human genome. The data archives, which provide the link between gene expression and eQTL according to ethnic populations, have been established and made freely available. Moreover, we also observed ancestry information enriched in eQTL. Finally, in applications, our results illustrate that eQTL provide crucial information not only for disease mechanism in medical genetics but also for population ancestry in population genetics as well as for adverse drug reactions, responses, and biotransformation in pharmacogenetics. Furthermore, integrating s-eQTL and g-eQTL increases the proportion of variability explained and the testing accuracy of prediction.

Performing PLS analysis identified the most substantial PLS factors that simultaneously explain both the variation of multiple response variables and the variation of multiple explanatory variables as much as possible. In this study, we investigated eQTL by using a gene-based PLS, where the response variables were transcripts in a T-gene and the explanatory variables were SNPs in an S-gene. Our method can jointly model multiple transcript probes and multiple SNP markers in a gene-based concept. Conventionally, the simple linear regression analysis that considers single transcript and single SNP marker is still most frequently used for a mapping of eQTL. It is well documented that PLS analysis outperforms the simple or multiple linear regression analysis, particularly for the following 2 situations: 1) when the number of observations (*n*) is smaller than the number of variables (*p*) (i.e., a problem of small *n* and large *p*); and 2) when co-linearity exists in explanatory variables. In practice, these situations occur in genome-wide eQTL mapping studies.

In this gene-based eQTL mapping study, the transcript expression levels of a T-gene were correlated with SNPs on an S-gene through a method of gene-based PLS. Gene-based eQTL mapping for g-eQTL is reduced to SNP-based eQTL mapping for s-eQTL if the transcript expression levels of genes were correlated with a single SNP in PLS analysis. In a comparison of SNP-based eQTL and gene-based eQTL mapping using PLS, these 2 types of eQTL mapping have their respective merits and the results are complementary. First, the SNP-based PLS identified more cis-acting regulators than the gene-based PLS did; 1,077 and 706 cis-acting genes were identified by the SNP-based PLS and gene-based PLS, respectively. However, a number of cis-regulators can be identified only by using the gene-based PLS; 136 cis-acting genes (75 in YRI, 56 in CEU, 55 in CHB, and 46 in JPT) could not be identified by using only SNP-based PLS. Second, SNP-based eQTL mapping is particularly suited for identifying ancestry-predictive eQTL that carry population-specific alleles for distant ethnic groups such as YRI and CEU. In this situation, a gene-based eQTL mapping can also be used to identify the ancestry-informative eQTL, but also inevitably includes other SNPs in the same gene. However, if the goal is to identify the AIMs of closely related populations, population-specific alleles in eQTL might not be available. In this case, when we can combine the eQTL from both s-eQTL and g-eQTL, the test accuracy for sample classification is higher than the results based only on s-eQTL or g-eQTL.

In this study, we identified the ancestry-informative eQTL associated with adverse drug reactions and drug responses. We identified rs1045642 (C3435T) on *ABCB1* as an ancestry-informative eQTL associated with adverse drug reactions and drug responses; Rs1045642 is a *C*/*T* polymorphism with an ancestral allele *C*. The allele frequency of the ancestral allele varied significantly across populations (*P* = 2.13E-13, using Fisher’s exact test); the allele frequencies in YRI, CEU, CHB, and JPT were 0.892, 0.458, 0.600, and 0.511, respectively. Previous studies have observed that this SNP associated with nortriptyline-induced postural hypotension in patients treated for major depression [101] and morphine pain relief in cancer patients [102].

The *ABCB1* gene encodes a P-glycoprotein. Activating this protein blocks drugs from entering the brain from the blood and also reduces the transportation of drugs out of the brain across the blood-brain barrier. Patients who carry the *TT* genotype tend to have lower gene expression of *ABCB1*, which reduces the activity of the encoding p-glycoproteins. Therefore, a reduced p-glycoprotein activity results in a relative accumulation of nortriptyline in the brain [101], thereby increasing the risk of postural hypotension in patients with treated with nortriptyline. However, suppression of p-glycoproteins also results in the accumulation of morphine in the brain, thereby enhancing the drug’s analgesic effects and pain relief [102].

Another ancestry-informative eQTL that was identified to be associated with drug response was rs20455 (Trp719Arg) on the kinesin family member 6 (*KIF6*) gene located on the chromosome 6p21.2. Rs20455 is a *C/T* polymorphism, where *C* is the ancestral allele. The frequency of the ancestral allele *C* dramatically varies according to populations (*P* = 2.76E-23, using Fisher’s exact test); the allele frequencies in YRI, CEU, CHB, and JPT are 0.931, 0.358, 0.556, and 0.400, respectively. Previous studies have revealed that rs20455 is associated with myocardial infarction [103] and coronary heart disease [103-105]. The missense change in the 719th codon from *CGG* to *TGG* alters the encoded amino acid to change from arginine (Arg) to tryptophan (Trp). People who carry the *C* allele(s) (719Arg) of rs20455 tend to have an increased risk of coronary heart diseases [103-106] relative to the *T* allele (Trp719). Our results indicated that people of African descents have a considerably higher frequency of the *C* allele of rs20455 than people of other populations do. This result is consistent with the high prevalence of cardiovascular diseases in populations of African-descent; for example, a previous survey revealed that the risk, prevalence, and mortality rates for cardiovascular disease are higher in African Americans than in Americans of other ethnic ancestries [107,108]. In addition, cardiovascular disease patients who carry the *C* allele(s) of rs20455 have an improved response to statin drugs such as pravastatin and atorvastatin, but no improvement was observed in non-carriers [103,105,106,109].

How *KIF6* influences the risk of cardiovascular disease and pharmacoresponse in statin drugs remains unclear. Several hypothetical mechanisms were proposed and await further investigation. For example, a missense substitution of the *C* allele of rs20455 with the *T* allele results in an amino acid change from Arg to Trp. The change might influence the affinity for transported cargo molecules and/or the motor activity of the *KIF6* protein and then influence the risk of cardiovascular disease and pharmacoresponse to statin [110].

This study also identified the eQTL that regulates the drug biotransformation genes [91,92]. Spastic paraplegia 7 (*SPG7*) was simultaneously identified as a cis-regulated biotransformation gene in all the HapMapII populations. This gene encodes a mitochondrial metalloprotease protein and was observed to be associated with docetaxel and thalidomide toxicities [111]. Transporter 2, ATP-bing cassette, sub-family B (*TAP2*) is a cis-regulated gene in CEU, CHB, and JPT, and Solute carrier family 7 (*SLC7A7*) is a cis-regulated gene in CHB and JPT. These 2 genes play a role in drug transportation [91,92]. Cytochrome P450, family 4, subfamily F, polypeptide 2 (*CYP4F2*) was identified as a cis-regulated gene in CEU and CHB. This gene belongs to the cytochrome P450 superfamily of enzymes, which is well known as the most crucial system of drug metabolism and bioactivation [112]. A previous study showed that *CYP4F2* can alter the required warfarin dose [113].

This study can be extended to several directions. First, this study analyzed the gene expression and SNP data from microarray experiments. With the advent of sequencing technology, the number of gene expression and SNP markers will dramatically increase in the future and researchers will be able to analyze more data from RNA sequencing and DNA sequencing experiments to gain more detailed information regarding gene regulation. However, we foresee that a sequencing-based eQTL mapping study will require intensive demands regarding computational facilities and time, and a substantial amount of space for recording the relations of gene expression and eQTL. An efficient algorithm for PLS will be crucial in this type of data analysis and a well-designed data warehouse will be required for storing the enormous amount of eQTL relations in the human genome.

Second, the type of biological specimen used in experiments might influence gene expression. This study provides a proof-of-concept method for eQTL mapping. The current results are still limited by the use of a single cell type, lymphoblastoid cell lines (LCL). LCL-based eQTL studies were recognized as essential materials for studying eQTL in providing the genetic mechanism of gene regulation [114]. However, because of the tissue-specific feature of eQTL, the findings of this study might not be applicable to other tissues and organs. We plan to extend our study to other types of tissue by analyzing data from the GTEx project [32]. It is worthwhile to compare the results from using LCL and other types of tissue sample and discuss the tissue-specific and tissue-shared patterns of gene regulation.

Third, the eQTL identified in this study might be limited by the small to moderate number of samples and ethnic populations available in the HapMapII project; therefore, the results should be further examined using additional samples from additional populations and require increased biological verifications. Currently, we are studying the relation of gene expression and eQTL of additional samples from additional populations by analyzing the data of the International HapMapIII Project, which contains 1,184 samples from 11 global populations [5,115].

Fourth, this study employed a set-based method of eQTL mapping. The gene is a natural and plausible unit, but not the only unit. We will extend our gene-based concept to incorporate the pathway information from Kyoto Encyclopedia of Genes and Genomes [116] and the gene ontology information of from the Gene Ontology project [117] for set-based eQTL mapping. The results will include insights into the regulatory network of the human genome and reveal more clues regarding disease etiology and population evolution.

Finally, this study only discussed mRNA regulation. To thoroughly understand regulation mechanisms, other mechanisms of gene regulation such as micro RNA and long non-coding RNA regulation, and epigenetic mechanisms such as DNA methylation, histone modification, and chromatin remodeling must be discussed.

### Availability and requirements

**Project name:** Gene-based mapping of expression quantitative trait loci

**Project home page:**http://www.stat.sinica.edu.tw/hsinchou/genetics/eQTL/HapMapII.htm

**Operating system:** MS Windows®

**Programming language:** SAS macro

**Data archive:** Excel

**Other requirements:** No

**Any restrictions to use by non-academics:** Citation

## Appendix

### The method of eQTL mapping, using a gene-based PLS analysis

Here we describe the procedure for implementing an eQTL mapping method, using a gene-based PLS. Without loss of generality, we discuss the case of a T-gene containing *t* transcripts and an S-gene containing *s* SNPs. In notation, matrix ***T*** denotes an *n* by *t* matrix of transcript data, where the element in the *i*th row and *j*th column is the expression level of the *j*th transcript of the *i*th individual. Matrix ***S*** denotes an *n* by *s* matrix of SNP data, where the element in the *i*th row and *j*th column takes a value of 0, 1, or 2 for genotype *AA*, *Aa*, or *aa*, respectively, for the *j*th SNP of the *i*th individual.

PLS is used to identify a number of factors that can explain the variation of response variables (***T***) and explain the variation of explanatory variables (***S***) as much as possible [118]. In general, the SNP matrix ***S*** and transcript matrix ***T*** can be expressed as a linear regression model of latent factors ***F***_***S***_ (dimensions: *n* by *r*) and ***F***_***T***_ (dimension: *n* by *r*) respectively as follows:

(A1) $$S=F_{S}L_{S}+E_{S}$$

(A2) $$T=F_{T}L_{T}+E_{T}$$

where ***L***_***S***_ (dimensions: *r* by *s*) and ***L***_***T***_ (dimensions: *r* by *t*) are loading matrices and ***E***_***S***_ (dimensions: *n* by *s*) and ***E***_***T***_ (dimensions: *n* by *t*) are error terms. Consider that ***F***_***T***_ can be rewritten as a linear model of ***F***_***S***_ as follows:

(A3) $$F_{T}=F_{S}L_{F}+E_{F}$$

where ***L***_***F***_ (dimensions: *r* by *r*) is a new loading matrix and ***E***_***F***_ (dimensions: *n* by *r*) is a new error term. From Equations (A2) and (A3), we obtain

(A4) $$\begin{array}{l} T=\left(F_{S}L_{F}+E_{F}\right)L_{T}+E_{T}\\ \;=F_{S}\left(L_{F}L_{T}\right)+\left(E_{F}L_{T}+E_{T}\right) \end{array}$$

From Equations (A1) and (A4), we obtain

$$\begin{array}{l} T=\left(S–E_{S}\right)\;{\left(L_{S}\right)}^{-1}\left(L_{F}L_{T}\right)+\left(E_{F}L_{T}+E_{T}\right)\\ \;=S\left[{\left(L_{S}\right)}^{-1}\left(L_{F}L_{T}\right)\right]\\ \;+\left[\left(E_{F}L_{T}+E_{T}\right)–E_{S}{\left(L_{S}\right)}^{-1}\left(L_{F}L_{T}\right)\right]\\ \;=SB_{\mathrm{PLS}}+E_{\mathrm{PLS}} \end{array}$$

where ***B***_**PLS**_ = (***L***_***S***_)^−1^ (***L***_***F***_***L***_***T***_) and ***E***_PLS_ = (***E***_***F***_***L***_***T***_ + ***E***_***T***_) – ***E***_***S***_ (***L***_***S***_)^−1^ (***L***_***F***_***L***_***T***_) are the regression coefficient matrix and error matrix in the PLS prediction model, respectively.

Basically, factors ***F***_***S***_ = ***S W***_***S***_ and ***F***_***T***_ = ***T W***_***T***_ are linear combinations of SNP variables and transcript variables, where the weight values ***W***_***S***_ and ***W***_***T***_ in the linear combinations can be calculated iteratively by maximizing the covariance of ***F***_***S***_ and ***F***_***T***_. The maximization procedure can be performed using a nonlinear iterative partial least squares algorithm. First, an initial value of ***W***_***S***_ is assigned to calculate ***F***_***S***_ = ***S W***_***S***_. Second, we regress ***S*** on ***F***_***S***_ and regress ***T*** on ***F***_***S***_ to obtain an estimate of the ***S***-loading matrix ***L***_***S***_ in Equation (A1) and ***T***-loading matrix ***L***_***F***_***L***_***T***_ in Equation (A4), respectively. Third, the procedure is iterated to obtain the first PLS factor ***F***_***S***_ to maximize the covariance of ***F***_***S***_ and ***F***_***T***_. Fourth, we deflate the ***S*** and ***T*** matrices by using ***S*** - ***F***_***S***_***L***_***S***_ and ***T*** - ***F***_***S***_ (***L***_***F***_***L***_***T***_), respectively. Finally, the ***S*** and ***T*** matrices are replaced with the deflated matrices ***S*** - ***F***_***S***_***L***_***S***_ and ***T*** - ***F***_***S***_ (***L***_***F***_***L***_***T***_), respectively, in the first 3 steps to extract the next PLS factor.

## Abbreviations

ADR: Adverse drug reaction; AIM: Ancestry-informative marker; BIASLESS: Biomarkers Identification and Samples Subdivision; CEU: CEPH Utah residents; CHB: Han Chinese in Beijing; eQTL: Expression quantitative trait loci; GEO: Gene Expression Omnibus; g-eQTL: Gene-based eQTL; HWE: Hardy-Weinberg Equilibrium; JPT: Japanese in Tokyo; LCL: Lymphoblastoid cell line; MNM: Multivariate nonparametric method; PLS: Partial least squares; PRESS: Predicted residual sum of squares; s-eQTL: SNP-based eQTL; S-gene: Sequence-level gene; SNP: Single nucleotide polymorphism; T-gene: Transcript-level gene; YRI: Yoruba in Ibadan.

## Competing interests

The authors declare that they have no competing interests.

## Authors’ contributions

HCY conceived and coordinated the study, interpreted the results, and prepared and revised the manuscript. CWL and CWC analyzed the data, constructed the eQTL data archives, and developed a SAS macro with HCY. CWC investigated the eQTL associated with population ancestry and pharmacogenetics together with HCY. JJC suggested the use of PLS and contributed to the discussion. All the authors have read and approved the final manuscript.

## Supplementary Material

Additional file 1 A SAS macro for eQTL mapping, using a gene-based PLS analysis. Click here for file

Additional file 2 **Title: Example of gene expression data – A file contains gene expression data from 7 T-genes in which each of them has 4 transcripts.** Description: This SAS data file contains 315 rows (45 individuals “times” 7 T-genes) and 6 columns. The first two columns are the names of T-gene and the names of people followed by the gene expression values of the 4 transcripts in order. Click here for file

Additional file 3 **Title: Example of genotype data – A file contains genotype data from 43 S-genes in which each of them contains 28 SNPs.** Description: This SAS data file contains 1,935 rows (45 people “times” 43 S-genes) and 30 columns. The first 2 columns are the names of S-gene and the names of people followed by their genotypic values (0, 1, or 2) for 28 SNPs in order. Click here for file

Additional file 4 **Title: Data archive 1 - Summary of the regulatory S-genes (g-eQTL) for each T-gene from our gene-based PLS analysis according to population.** Description: This Excel file provides a table that summarizes the regulatory S-genes (g-eQTL) for each T-gene from our gene-based PLS analysis by population. The table consists of 3 components and each row represents a T-gene. The first component provides “T-gene name”: each gene is named by its gene symbol followed by an Entrez gene ID. For example, the gene symbol and gene ID of the first gene in this table are 2′-PDE and 201626. The second component provides the “data availability” of a gene expression and SNP: “1” indicates the data is available and “NA” indicates the data is not available in the g-eQTL mapping. The third component provides the g-eQTL names that regulated T-genes through a cis- or trans-regulatory mechanism according to population. For each population, the first column records if a cis-acting regulation was occurred in the T-gene (“1” indicates a cis-acting relation was identified and “NA” indicates there was no cis-acting relation). The second column summarizes all the trans-acting S-genes that regulate a T-gene. Each S-gene is named by its gene symbol followed by an Entrez gene ID, and multiple S-genes are separated by commas. Click here for file

Additional file 5 **Title: Data archive 2 - Summary of T-genes regulated by each S-gene from our gene-based PLS analysis according to population.** Description: This Excel file provides a table that summarized the T-genes regulated by each S-gene from our gene-based PLS analysis by population. The table consists of 3 components and each row represents an S-gene. The first component provides the “S-gene name” and “Probe_Set”: each S-gene is named by its gene symbol followed by an Entrez gene ID. All the SNPs contained in each S-gene are listed in the “Probe_Set” column; multiple SNPs are separated by commas. The second component provides the “data availability” of gene expression and SNP, and “1” indicates that the data is available and “NA” indicates it is not available in the g-eQTL mapping. The third component provides the names of T-genes regulated by S-genes through a cis- or trans-regulatory mechanism according to population. For each population, the first column records whether a cis-acting regulation occurred in an S-gene (“1” indicates a cis-acting relation was identified and “NA” indicates there was no cis-acting relation). The second column summarizes all trans-acting T-genes regulated by an S-gene. Each T-gene is named by its gene symbol followed by an Entrez gene ID, and multiple T-genes are separated by commas. Click here for file

Additional file 6 **Title: Data archive 3 - Summary of T-genes regulated by each eQTL on chromosome 1 from our SNP-based PLS analysis according to population.** Description: This Excel file provides a table that summarized the T-genes regulated by each eQTL from our SNP-based PLS analysis by population. The table consists of 3 components and each row represents an eQTL. The first component provides the “SNP ID (Probe_Set)”, “Gene symbol”, and “Gene ID”: each eQTL is named by the SNP probe set. The gene symbol and Entrez gene ID of the gene where an eQTL was located are provided. The second component provides the “data availability” of the SNPs: “1” indicates that the data is available and “NA” indicates it is not available in the g-eQTL mapping. The third component provides the names of T-genes regulated by eQTL through a cis- or trans-regulatory mechanism according population. For each population, the first column records whether a cis-acting regulation occurred in an S-gene (“1” indicates a cis-acting relation was identified and “NA” indicates there was no cis-acting relation). The second column summarizes all the trans-acting T-genes regulated by an eQTL. Each T-gene is named by its gene symbol followed by an Entrez gene ID, and multiple T-genes are separated by commas. Because of the constraint of file size, only eQTL on chromosome 1 is provided in this Excel file. The results regarding eQTL on other chromosomes are accessible at the website (http://www.stat.sinica.edu.tw/hsinchou/genetics/eQTL/HapMapII.htm). Click here for file
