# Supplementary material for: Applying genome-wide gene-based expression quantitative trait locus mapping to study population ancestry and pharmacogenetics
Source: BMC Genomics. 2014 Apr 29;15:319. doi: 10.1186/1471-2164-15-319 (PMC4236814; doi:10.1186/1471-2164-15-319)
Supplement: Additional file 1 — A SAS macro for eQTL mapping, using a gene-based PLS analysis. [file 1471-2164-15-319-S1.doc]

**Additional file 1: A SAS macro for eQTL mapping, using a gene-based PLS analysis**

%macro gbPLS(Tgene=, Sgene=, nProbe=, nSNP=, Out=);

/*******************************************************************

Tgene: T-genes' data contained columns "Tgene", "Ind", "Probe1", "Probe2" ...

Sgene: S-genes' data contained columns "Sgene", "Ind", "SNP1", "SNP2" ...

nProbe: number of probes in T-genes

nSNP: number of SNPs on S-genes

Out: output directory

*******************************************************************/

libname OUT "&Out.";

/* data appending recursively */

%macro APPEND(y=, n=);

%do r=1 %to &n.;

proc append data=&y. base=&y. force;

run;

%end;

%mend;

/* data sorting */

proc sort data=&Tgene. out=T;

by Tgene Ind;

run;

proc sort data=&Sgene. out=X;

by Sgene Ind;

run;

/* number of T-genes */

proc freq data=T;

table Tgene;

ods output OneWayFreqs = FREQ;

run;

data FREQ;

set FREQ;

call symput('y', trim(left(_n_)));

call symput('y'||trim(left(_n_)), trim(left(Tgene)));

run;

/* number of S-genes */

proc freq data=X;

table Sgene;

ods output OneWayFreqs = FREQ;

run;

data FREQ;

set FREQ;

call symput('x', trim(left(_n_)));

run;

%let n= %sysfunc(ceil(%sysevalf(%sysfunc(log(&x.))/%sysfunc(log(2)))));

%put &n;

%put &y;

%put &x;

/* analysis by T-genes */

%do i=1 %to &y.;

data Y;

set T(where=(Tgene="&&y&i.."));

run;

%APPEND(y=Y, n=&n.);

/* gene-based PLS */

data M;

merge Y X;

if Sgene~="";

proc pls method = pls cv = split cvtest (seed = 12345) missing = EM(maxiter = 1000) details;

model Probe1-Probe&nProbe. =SNP1-SNP&nSNP./solution;

by Tgene Sgene;

ods output ModelInfo = OUT.&&y&i.._MI_cv;

ods output PercentVariation = OUT.&&y&i.._PV_cv;

ods output CenScaleParms = OUT.&&y&i.._CSP_cv;

ods output CVResults = OUT.&&y&i.._CV;

ods output ResidualSummary = OUT.&&y&i.._RS;

proc pls data=TMP.M method = pls missing = EM(maxiter = 1000) details;

model Probe1-Probe&nProbe. =SNP1-SNP&nSNP./solution;

by Tgene Sgene;

ods output ModelInfo = OUT.&&y&i.._MI;

ods output PercentVariation = OUT.&&y&i.._PV;

ods output CenScaleParms = OUT.&&y&i.._CSP;

run;

%end;

%mend;

libname Example "c:\HapMap_PLS_eQTL\";

%gbPLS(Tgene=Example.Y_4_probe, Sgene=Example.X_28_SNP, nProbe=4, nSNP=28, Out=c:\HapMap_PLS_eQTL);
